# Predictive impact of PI-RADS 3 lesion volume/total prostate volume ratio in prostate cancer diagnosis in biopsy-naïve patients

**DOI:** 10.55730/1300-0144.6103

**Published:** 2025-10-05

**Authors:** Emrah ÖZSOY, Musab Ali KUTLUHAN, Emre TOKUÇ, Rıdvan KAYAR, Samet DEMİR, Kaan MERİÇ, Metin İshak ÖZTÜRK

**Affiliations:** 1 Department of Urology, Ünye Çakırtepe Hospital, Ordu, Turkiye; 2 Department of Urology, Faculty of Medicine, Ankara Yıldırım Beyazıt University, Ankara, Turkiye; 3 Department of Urology, Haydarpaşa Numune Training and Research Hospital, Health Sciences University, İstanbul, Turkiye; 4 Department of Urology, Van Training and Research Hospital, Health Sciences University, Van, Turkiye; 5 Department of Radiology, Medistate Hospital, Beykoz University, İstanbul, Turkiye

**Keywords:** Prostate cancer, multiparametric MRI, PI-RADS score, PI-RADS-3, PI-RADS percent

## Abstract

**Background/aim:**

To assess the potential of the ratio between PI-RADS 3 lesion volume and total prostate volume as a predictive parameter for guiding the decision to perform a biopsy in patients presenting with PI-RADS 3 lesions on multiparametric prostate magnetic resonance imaging (mpMRI).

**Materials and methods:**

A total of 749 patients who underwent mpMRI due to suspected prostate cancer between January 2014 and August 2023 were scanned. Based on predefined inclusion and exclusion criteria, 308 patients were included. Age, total prostate-specific antigen (PSA) value, prostate volume measured in mpMRI, mpMRI result, PI-RADS 3 lesion volume, and biopsy results were collected. The PI-RADS 3 ratio was calculated as PI-RADS 3 lesion volume/total prostate volume. PSA density (dPSA) was calculated. The patients were categorized according to their biopsy results as benign or malignant (subclassified by Gleason group grade), and the two groups were compared.

**Results:**

The average PI-RADS 3 ratio was 0.032 ± 0.002. There were 230 (74.7%) patients in the benign group and 78 (25.3%) patients in the malignant group. There was a statistically significant difference detected in average prostate volumes (p < 0.001), dPSA values (p = 0.001), and PI-RADS 3 ratios (p < 0.001). The receiver operating curve analysis of PI-RADS 3 ratio indicated an area under the curve of 0.643 ± 0.037. The optimal cut-off point was 0.026 with a sensitivity of 58.97% and a specificity of 66.96%. A positive, albeit weak, statistically significant relationship was found between PIRADS-3 ratios and dPSA values (rs ρ = 0.261 and p < 0.001).

**Conclusion:**

PI-RADS 3 ratio may serve as an auxiliary clinical parameter alongside age, dPSA, and lesion volume alone in identifying more refined candidates for biopsy in the goal of patient care individualization.

## 1. Introduction

Multiparametric prostate magnetic resonance imaging (mpMRI) is currently recommended for all biopsy-naïve patients with suspected prostate cancer (PCa) prior to undergoing a biopsy procedure [1]. The main rationale of utilizing mpMRI in this patient group is to maximize the efficiency of biopsy procedures while simultaneously reducing the number of unnecessary biopsies [2]. According to Prostate Imaging-Reporting and Data System version 2.1 (PI-RADS v2.1), which is the most widely adopted framework for interpreting mpMRIs today, the likelihood of detecting PCa is low in lesions categorized as PI-RADS 1 or 2, whereas lesions classified as PI-RADS 4 or 5 are associated with a high probability of malignancy [3,4]. PI-RADS category 3 lesions, however, represent an indeterminate group with an equivocal probability of malignancy, posing a diagnostic challenge for both clinicians and patients in differentiating between benign and malignant pathology [5].

PI-RADS 3 lesions are considered “the gray zone” when the intended use of mpMRI in clinical practice is taken into account. Since it has been shown that the incidence of clinically significant prostate cancer (csPCa) in PI-RADS 3 lesions may be as high as 25%, the biopsy decision of this patient group relies on additional clinical factors such as digital rectal examination, age, tumor volume, and prostate-specific antigen (PSA) density [6,7]. In this regard, Rico et al. suggested that the detection rate of csPCa is significantly higher in patients with PI-RADS 3 lesions, exhibiting a tumor volume of >0.5 mL and a PSA density of >0.15, and unnecessary biopsies may be prevented up to 83.8% in this patient group [8].

The PI-RADS ratio refers to the proportion of lesion volume, as identified by mpMRI, relative to the total prostate volume. This parameter has been reported to possess predictive value for adverse pathological outcomes following prostatectomy [9]. Additionally, both absolute tumor volume and tumor volume percentage have been employed in the literature as prognostic indicators for biochemical recurrence, based on the evaluation of pathology specimens obtained after radical prostatectomy [10].

The aim of this study is to assess the predictive ability of PI-RADS 3 volume to total prostate volume ratio as an auxiliary clinical parameter in the decision-making process for biopsy or follow-up in patients with PI-RADS 3 lesions, with the potential to subsequently increase the efficiency of biopsies by avoiding unnecessary interventions.

## 2. Materials and methods

### 2.1. Patient selection and study design

Ethics committee approval for this study was obtained from the Institutional Review Board of Haydarpaşa Numune Training and Research Hospital Clinical Research Ethics Committee on November 6, 2023 (HNEAH-KAEK 2023/KK/200). This retrospective study was initiated by reviewing the records of 749 patients who presented to our tertiary referral center between January 2014 and August 2023. These patients underwent mpMRI due to suspected prostate cancer, based on elevated PSA levels or abnormal findings on digital rectal examination, and subsequently underwent prostate biopsy.

Following the application of predefined inclusion and exclusion criteria, 308 patients were included in the final analysis. The medical records of these patients were reviewed to extract relevant data, including age, total PSA level (ng/mL), prostate volume measured via mpMRI (mL), mpMRI findings, PI-RADS 3 lesion volume (mL), and biopsy results (Gleason score and Gleason grade group). Prostate and lesion volumes were calculated using the ellipsoid formula (length × width × height × π/6) [11]. All PSA measurements were conducted in the same laboratory within the institution to ensure consistency.

PSA density (dPSA) was calculated using the formula: total PSA/total prostate volume. The PI-RADS 3 ratio was defined as the PI-RADS 3 lesion volume divided by the total prostate volume. Both values were incorporated into the dataset for further analysis.

All prostate biopsies were performed transrectally under ultrasound guidance. Imaging was conducted using a 1.5 Tesla MRI scanner, and all PI-RADS assessments were carried out by the same radiologist. Lesions originally evaluated according to PI-RADS version 1 were reinterpreted using PI-RADS version 2 for consistency.

According to the prostate biopsy results, two groups were created, with 78 patients in the malignant group and 230 patients in the benign group, and the data were compared statistically.

### 2.2. Inclusion and exclusion criteria

Patients between the ages of 40 and 75, who had a single PI-RADS 3 lesion detected as a result of mpMRI and who had a prostate biopsy for the first time, were included in the study. The exclusion criteria of our study are being under the age of 40 or over the age of 75, having previously prostate biopsy, having a non-PI-RADS 3 lesion or multiple lesions detected by mpMRI, being on 5-a reductase inhibitor or testosterone therapy, previous benign or malignant prostate surgery history, having a history of radiotherapy to the pelvic area for any reason, having a first-degree relative with prostate cancer, having acute or chronic prostatitis history, and having chronic prostatitis detected as a result of biopsy.

### 2.3. Statistical analyses

Descriptive statistics were calculated and presented as “Mean ± SEM” (standard error of the mean [SEM]) or “Median (Minimum–Maximum)” for continuous variables, and n (%) for categorical variables. Prior to hypothesis testing, data were checked with the Shapiro–Wilk test for normality and the Levene test for homogeneity of variances as parametric test assumptions. The Mann–Whitney *U* test was used to compare the groups (malignant vs. benign), taking into account the fulfillment of parametric test assumptions. Spearman’s rank correlation test was used to assess correlations among variables. Receiver operating curve (ROC) analysis was used to evaluate the diagnostic efficacy of PI-RADS percentage values, and the area under the curve (AUC) with standard error and 95% confidence intervals (CIs) was calculated. Youden’s J statistic index was used to determine the decision threshold of the PI-RADS percentage in diagnosing malignancy. Sensitivity, specificity, and accuracy values were calculated for the proposed cut-off value, using pathological assessment as the gold standard. The DeLong Method was used for calculating standard errors. A cut-off value of 0.15 for dPSA was used for the classification of malignancy prediction. The McNemar test was used for comparison of sensitivities and specificities using the DTCompPair package for R [12]. All statistical analyses were performed with STATA 18 and RStudio (v.4.2.1; R Core Team, 2021). A p-value of ≤ 0.05 was considered to be statistically significant.

## 3. Results

The average age of the patients in the study was 62.72 ± 0.35 years. The mean prostate volume was 74.42 ± 1.96 mL. The descriptive characteristics of the 308 patients included in the study are summarized in Table 1.

The average PSA value was 6.86 ± 0.2 ng/mL, and the mean lesion volume was 2.05 ± 0.13 mL. The mean PSA density (dPSA) was 0.11 ± 0.01, and the mean PI-RADS 3 ratio was 0.032 ± 0.002. Based on the prostate biopsy results, 230 patients (74.7%) were classified into the benign group, while 78 patients (25.3%) were placed in the malignant group. Within the malignant group, the distribution according to the Gleason group grade (GG) classification was as follows: 52 patients (16.9%) were classified as GG1, 14 patients (4.5%) as GG2, nine patients (2.9%) as GG3, two patients (0.6%) as GG4, and one patient (0.3%) as GG5.

Statistical comparison between benign and malignant groups is detailed in Table 1. The average prostate volume in the benign group was 79.33 ± 2.29 mL, whereas it was significantly smaller in the malignant group, with a value of 59.92 ± 3.32 mL (p < 0.001). No significant difference was observed in PI-RADS 3 lesion volumes between the two groups. However, PSA values were found to be higher in the benign group (p = 0.116 and p = 0.036). The mean PSA density (dPSA) in the malignant group was 0.12 ± 0.01, compared to 0.10 ± 0.01 in the benign group, and this difference was statistically significant (p = 0.001). When comparing the PI-RADS 3 ratios between the two groups, the malignant group exhibited significantly higher ratios (p < 0.001).

ROC curve analysis with PI-RADS 3 ratios is shown in Figure. AUC was determined to be 0.643 ± 0.037. According to the Youden J index, the optimal cut-off point is 0.026. The sensitivity and specificity comparison made in the study by taking the dPSA cut-off value as 0.15 and the PI-RADS 3 ratio cut-off value as 0.026 is given in Table 2. While the sensitivity for dPSA is 20.5% and the specificity is 87.4%, for the PI-RADS 3 ratio, the sensitivity is 58.97% and the specificity is 66.96%.

The correlation between PSA density (dPSA) and the PI-RADS 3 ratio was assessed using Spearman’s rho coefficient test, which yielded a correlation coefficient of rs ρ = 0.261 and a p-value of <0.001. This indicates a positive, albeit weak, statistically significant relationship between PI-RADS 3 ratios and dPSA values.

## 4. Discussion

In clinical practice, mpMRI is an aiding imaging modality that assists the clinician in suspecting PCa with significant side-advantages secondary benefits such as reducing unnecessary biopsies and therefore preventing associated side effects, mitigating the risk of PCa overdiagnosis, monitoring patients in active surveillance, or evaluating local recurrence in patients after radical prostatectomy or radiotherapy. In the 2017 PROMIS study by Hashim et. al involving 740 biopsy-naïve patients, it was suggested that using mpMRI prior to transrectal ultrasound-guided biopsy (TRUS-bx) reduces unnecessary biopsy rates by 27%, reduces overdiagnosis by 5%, and increases the detection rate of csPCa by 18%, compared with TRUS-bx alone [13]. In another study, it was highlighted that mpMRI is more sensitive and specific than PSA, and could potentially be utilized as a screening test instead of PSA, raising some concerns about cost-effectiveness [14]. Following the impact of mpMRI shown in clinical studies, the American Urology Association (AUA) and European Association of Urology (EAU) guidelines strongly recommend mpMRI prior to biopsy [1,15]. Despite all these benefits of mpMRI, the management of patients with PI-RADS 3 lesions remains challenging. In this study, the PI-RADS 3 ratio was evaluated as a potential parameter to assist in decision-making for biopsy or follow-up in patients with PI-RADS 3 lesions, which are often considered “gray zones” in terms of malignancy risk.

In addition to the increasing popularity of mpMRI in recent years, the idea of using imaging to suggest that there is or is not a malignancy without tissue diagnosis has raised some questions and concerns regarding the reliability of this method. Pitfalls in imaging techniques, intra- and interreader variability, and PI-RADS 3 lesions are the main factors that reduce the diagnostic accuracy of mpMRI [16,17]. As a result of a systematic review and metaanalysis conducted by the EAU Prostate Cancer Panel in 2017, it was recommended that the biopsy decision should not be canceled without risk stratification in the patient group with negative mpMRI [18].

PI-RADS 3 lesions are challenging for the clinician when concluding the biopsy decision and planning the diagnosis and follow-up processes of especially in biopsy-naïve patients requires attentive elaboration. In a metaanalysis conducted by Hamoen et al. in 2014, it was determined that the sensitivity of mpMRI is 0.88 (95% CI 0.82–0.93) and specificity is 0.45 (95% CI 0.27–0.65) in lesions of PI-RADS 3 and above, while sensitivity is 0.66 (95% CI 0.54–0.76) and specificity is 0.76 (95% CI 0.63–0.85) in lesions of PI-RADS 4 and above [19]. Also, in a review conducted in 2017, the prevalence of PI-RADS 3 index lesion in the diagnostic work-up is suggested as significant, varying from 22–32% of men, and the prevalence of csPCa (GS ≥ 3 + 4) in PI-RADS 3 lesions varied from 16–21% [20].

Performing a biopsy on every patient with a PI-RADS 3 lesion can lead to overdiagnosis and increased complication rates. Additionally, following all patients in this group may result in missing approximately 20% of clinically significant prostate cancer (csPCa), as previously noted. Therefore, studies have focused on identifying auxiliary clinical factors such as age, PSA density (dPSA), and lesion volume that can assist in making biopsy or follow-up decisions for the PI-RADS 3 patient group [6,7,21]. As a result of the multiinstitutional collaborative study conducted by Drevik et al., using dPSA of 0.15 when deciding on biopsy in the PI-RADS 3 patient group reduced the csPCA skip rate from 20% to 13.6% [22]. However, in our study, the contribution of dPSA was not found to be significant. According to this study, in the diagnostic evaluation made by accepting the dPSA cut-off value as 0.15, sensitivity was 20.5% (95% CI 12.2–31.2) and specificity was 87.4% (95% CI 82.4–91.4). It was determined that the dPSA value of 62 (79.5%) of 78 patients with malignant pathology was <0.15. This suggests that relying solely on dPSA may not be effective for making biopsy or follow-up decisions in this patient group.

Another valuable clinical factor is lesion volume. In a 2017 study, Martorana et al. reported a direct proportionality between csPCa detection rates and lesion volume [23]. Based on this and similar studies, a subclassification of PI-RADS 3 lesions was introduced: PI-RADS 3a for lesions with a volume <0.5 mL and PI-RADS 3b for lesions with a volume >0.5 mL [8,23,24]. In our study, while the lesion volume in eight of the 78 patients with malignant pathology was <0.5 mL, the lesion volume in 204 of the 230 patients in the benign group was >0.5 mL. When considering lesion volume as an auxiliary clinical factor, it should be acknowledged that patients with smaller prostate sizes may have smaller lesion volumes, potentially leading to false-negative results. In addition to using auxiliary clinical factors alone in PI-RADS 3 lesions, algorithms have also been created to evaluate these factors together.[24].

In the study published by Yuk et al. in 2022, which included 2394 radical prostatectomized localized prostate cancer patients, it was stated that the tumor volume/prostate volume parameter obtained after measurements made in pathology specimens was an independent prognostic factor for short biochemical recurrence-free survival [25]. The literature also contains studies indicating that this parameter possesses predictive value in relation to recurrence, biochemical recurrence, and Gleason score upgrading following radical prostatectomy [10,26,27]. Taken together, these studies suggest that the tumor volume-to-prostate volume ratio may have initial prognostic utility at the diagnostic stage of prostate cancer.

The term “PI-RADS ratio” was introduced for the first time by Ratnani et al. in their 2022 study [9]. In this investigation, the authors evaluated the predictive value of the lesion volume to prostate volume parameter, as measured by mpMRI, for adverse pathological outcomes following surgery in patients with PI-RADS 3-4-5 3, 4 or 5 lesions. The study findings indicated that an increased PI-RADS ratio was significantly associated with a higher risk of adverse pathology.

In our study, we found that the mean PI-RADS 3 percentage in the malignant patient group was significantly higher than in the benign group (p < 0.001). As shown in the Figure, the result of the ROC curve analysis for the PI-RADS 3 ratio, the AUC is 0.643 ± 0.037. The optimal cut-off value for PI-RADS 3 ratio is >0.026. According to this cut-off value, sensitivity and specificity values for PI-RADS 3 ratio were determined as 58.97% and 66.96% as shown in Table 2. When the comparison between dPSA and PI-RADS 3 ratio in terms of diagnostic effect is evaluated, sensitivity is significant in favor of PI-RADS 3 ratio, while specificity is significant in favor of dPSA. The association between PI-RADS 3 ratio and dPSA was significantly positive, even though it was statistically weak. PI-RADS 3 lesions exhibiting a PI-RADS ratio below 0.026 are more likely to be benign. Conversely, in patients whose auxiliary diagnostic parameters—such as PSA density—do not independently indicate prostate cancer, a PI-RADS 3 ratio exceeding 0.026 may justify the recommendation for prostate biopsy or the implementation of a more stringent follow-up protocol.

Unfortunately, the AUC value in this result is <0.80, which is one of the limitations of our study. This result was thought to be due to the small size of the cohort. Additionally, the limited number of patients precluded the stratification of the PI-RADS 3 ratio by Gleason group grade within the malignant cohort, thereby restricting our ability to demarcate clinically significant prostate cancer (csPCa). Moreover, since only solitary PI-RADS 3 lesions were included, our findings cannot be generalized to patients with multiple lesions. Finally, only TRUS-bx is performed due to technical inadequacies. It should be noted that false-negative results are more likely with TRUS-bx when compared to MRI-fusion or in-bore biopsy techniques. In clinical practice, patients with negative TRUS-bx results but still suspicious for prostate cancer can be diagnosed with MRI-fusion or in-bore biopsy. Consequently, if our study had included patients who underwent MRI-fusion or in-bore biopsy, the assessment of the sensitivity and specificity of the PI-RADS 3 ratio could have been more robust. Moreover, since the PI-RADS 3 ratio is fundamentally an MRI-based parameter, inter- and intrareader variability may affect the results. In view of these limitations, multicentric and prospective studies with larger cohorts—particularly those capable of discriminating clinically significant prostate cancer—are warranted.

This study stands out in the literature as the first to investigate the PI-RADS 3 ratio as an auxiliary clinical factor in the PI-RADS 3 patient group. In addition to established parameters such as age, PSA density, and lesion volume used to guide biopsy or follow-up decisions, the PI-RADS 3 ratio may serve as an adjunctive measure, potentially reducing false-negative results associated with other factors. Although the PI-RADS 3 ratio is not currently employed as a standalone diagnostic parameter for prostate cancer, it holds promise for inclusion in future clinical and radiological nomograms and predictive models. Further large-scale studies are necessary to draw more definitive conclusions.

## Figures and Tables

**Figure:**
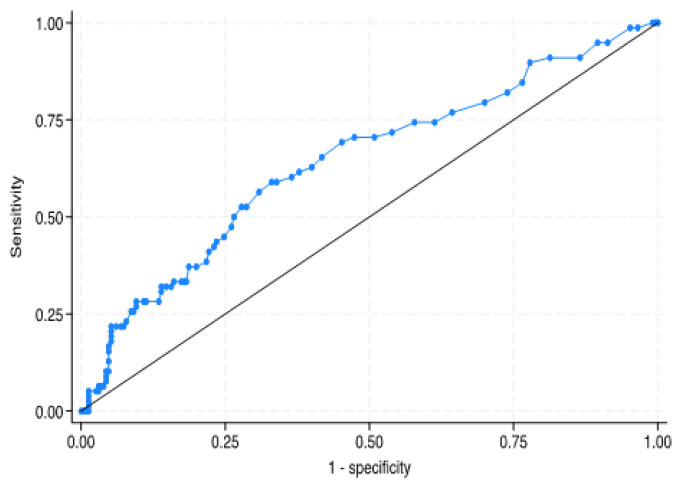
ROC curve analysis of PI-RADS percent (area under the curve = 0.6429).

**Table 1:** Descriptive analysis and statistical comparisons between benign and malignant groups.

	Malignant (n: 78)			Benign (n: 230)			
Variable	Mean ± SEM	Median (min–max)	SD	Mean ± SEM	Median (min–max)	SD	p
**Age**	61.78 ± 0.83	63 (45–75)	7.29	63.04 ± 0.38	63 (42–76)	5.69	0.313
**Volume (mL)**	59.92 ± 3.32	49.5 (16–133)	29.34	79.33 ± 2.29	72 (30–328)	34.76	<0.001
**PSA (ng/mL)**	6.25 ± 0.35	5.4 (1–18.5)	3.07	7.06 ± 0.24	6.2 (2–27)	3.58	0.036
**Lesion volume (mL)**	2.03 ± 0.16	1.65 (0.3–6.6)	1.4	2.06 ± 0.16	1.25 (0.1–21)	2.48	0.116
**dPSA (ng/mL/cc)**	0.12 ± 0.01	0.1 (0.03–0.78)	0.09	0.1 ± 0.01	0.08 (0.02–0.94)	0.1	0.001
**PI-RADS percent**	0.044 ± 0.005	0.031 (0.003–0.175)	0.04	0.028 ± 0.002	0.018 (0.001–0.3)	0.034	<0.001

SEM: standard error of mean, SD: standard deviation, PSA: prostate-specific antigen, dPSA: prostate-specific antigen density, PI-RADS: Prostate Imaging-Reporting and Data System.

**Table 2:** Statistical comparison between the diagnostic performance of dPSA and PI-RADS percent.

		Malignant (gold standard)					
		Benign		Malignant		Sensitivity (%) (95% CI)	Specificity (%) (95% CI)
Classification variable (cut-off)		*n*	*n (%)*	*n*	*n (%)*		
dPSA (0.15)	Benign	201	87.40%	62	79.50%	20.5	87.4
	Malignant	29	12.60%	16	20.50%	(12.2–31.2)	(82.4–91.4)
PI-RADS percent (0.026)	Benign	154	67.00%	32	41.00%	58.97	66.96
	Malignant	76	33.00%	46	59.00%	(47.3–70.01)	(60.5–73.01)
					***McNemar***	*23.68*	*30.26*
					***p-value***	*<0.001*	*<0.001*

dPSA: prostate-specific antigen density, PI-RADS: Prostate Imaging-Reporting and Data System.
